# Seismic response analysis of super-high-rise building structures with three-layer isolation systems

**DOI:** 10.1038/s41598-023-46207-8

**Published:** 2023-11-06

**Authors:** Yuan Zhang, Zhongfa Guo, Dewen Liu, Weiwei Sun

**Affiliations:** 1 College of Civil Engineering, Southwest Forestry University, Kunming, 650000 Yunnan China; 2 Rattana Bundit University, Mangu, 10700 Thailand

**Keywords:** Civil engineering, Natural hazards, Seismology

## Abstract

This paper proposes a triple-layer isolation device based on the single-story isolation and double-layer isolation. By establishing dynamic equilibrium equations and conducting earthquake response comparative analyses on the same super-tall building structure using three different forms of isolation: single-story isolation, double-layer isolation, and triple-layer isolation, it is found that the triple-layer isolation device, which adds an isolation layer on the basis of the double-layer isolation device, exhibits many different characteristics in terms of its damping mechanism, seismic response rules, etc., due to the differences in the position and quantity of the isolation layer. Rare earthquakes are equivalent to ASCE maximum considered earthquake or annual occurrence probability of 2%. The research shows that, under rare earthquake conditions, the isolation effect of the triple-layer isolation device in the super-tall frame-shear structure is better than that of the single-story isolation and double-layer isolation devices. Particularly, as the number of floors increases, the triple-layer isolation device can significantly reduce the seismic isolation support tension and compression stresses, inter-story displacement, single-story shear force, overturning moment, and floor acceleration of the high-rise building structure, and concentrate the lateral displacement of the structure on the three isolation layers, dissipating most of the seismic input energy.

## Introduction

In recent years, high-rise buildings have experienced rapid development worldwide, coupled with frequent seismic disasters. In order to meet the needs of building functional layout and seismic resistance, one or more isolation layers are often required, and such buildings have become a major trend in modern high-rise building development. Scholars at home and abroad have made useful explorations in the study of the seismic performance of multi-layer isolation systems for high-rise buildings. According to the different positions of isolation layers, there are many forms of isolation structures. Ou^1^ proposed a new type of structure called the large-displacement friction pendulum, in which the structural layer serves as the sliding surface, and the whole or part of the structure between the sliding surface serves as the sliding block. By varying the location and number of isolation layers, a large-displacement friction pendulum bottom isolation layer and a multi-layer isolation structure system were constructed. Wang et al.^2^ proposed a segmented isolation structure form for high-rise buildings with large height-to-width ratios based on laminated rubber bearings, and conducted numerical simulation and comparative analysis on the same high-rise building structure using three different forms of isolation: base isolation, single-story isolation, and segmented isolation. The results showed that the segmented isolation had a more obvious effect. Gao^3^ proposed a novel segmented seismic isolation system based on its technical sources and fundamental principles. They developed an energy-based passive control parameter analysis method starting from the principles of stochastic vibration theory and energy balance. Wu^4^ focused on high-rise frame structures and proposed a new hybrid passive control system that combines segmented seismic isolation with adjacent building connection dampers for energy dissipation. This system effectively mitigates wideband ground motions and has higher robustness and redundancy. Segmented isolation layers have been applied to high-rise buildings abroad^5,6^, while inter-story isolation (ISI) is suitable for retrofitting and combining different structural systems. Additionally, as the isolation layer moves from the foundation to higher floors, it can eliminate seismic gaps at the foundation and is suitable for mid-to-high-rise buildings. The configuration of multiple inter-story isolation (ISI) layers at different floors, referred to as multi-story isolation (MSI) here, has been shown to reduce maximum isolation drift without increasing the primary building response variables. This finding is significant because isolation drift has been identified as an indicator of adverse response modes. Some scholars such as Pan et al.^7^ considered a fixed configuration, which installed four isolation layers on specific floors of a 16-story building. Charmpis et al.^8^ implicitly treated the number of isolation layers and their positions as design variables to optimize the seismic performance of structures. In a single-objective optimization problem, they used the isolation drift of the base isolation layer as a constraint but did not consider the drift of upper isolation layers. Skandalos et al.^9^ 9proposed an inter-story isolation (ISI) configuration with multiple isolation layers, which brought a large design space and many possible isolation configurations and properties for controlled buildings. During strong earthquakes, the effectiveness of this system in further extending the building period surpasses that of classical isolation methods and mostly reduces single-story deflection. In order to reduce the displacement of the single-story, the experimental results from Hongkai Du's^10^ study suggest that a well-considered combination of soft limiter stiffness and reserved gap effectively restrains the isolated base displacement response, with minimal adverse impact on the superstructure. Han et al.^11^ conducted experimental research on the seismic performance of a steel frame base-isolated structure employing steel spiral spring limiters under near-fault ground motions. The results further elucidated the seismic influence of limiter stiffness and reserved gap size on the dynamic response of the isolation layer and superstructure. One of the main findings by Deringöl^12^ and his colleagues is that base-isolated buildings with passive damping devices respond satisfactorily when combined with appropriate design parameters.

The previous studies on multi-story base isolation devices mainly focused on double-layer base isolation structures, and there have been no studies by scholars on triple-layer isolation devices with more than two isolation layers. The damping mechanism, seismic response characteristics, and other aspects of triple-layer isolation devices differ from those of single-story isolation and double-layer base isolation structures, which may exhibit a series of new features. In this paper, a triple-layer isolation super high-rise frame-shear wall structure model was established using ABAQUS^13^ software to investigate the seismic response under earthquake excitation, and a comparative analysis was conducted with single-story isolation and double-layer isolation devices.

## The establishment of the dynamic equilibrium equation

### System model

**Figure 1:**
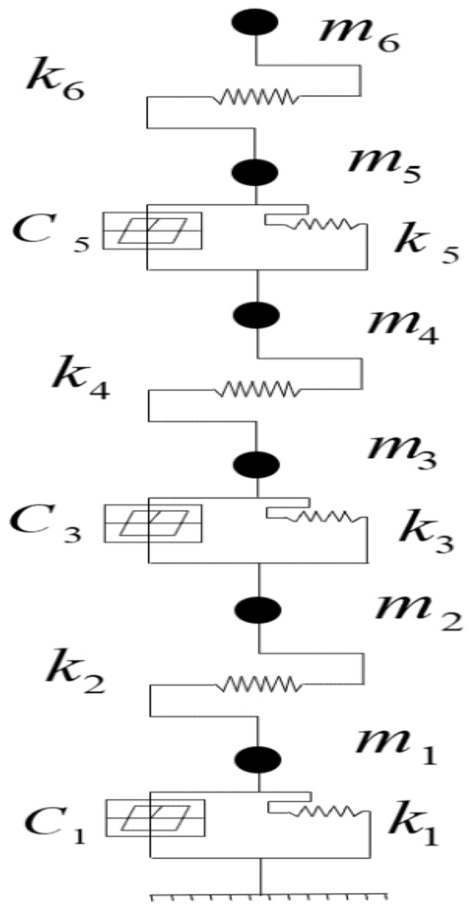
Particle model.

In order to better explain the mechanism of the triple-layer isolation device, a simplified mass-spring model was used to analyze the structure, as shown in Fig. 1. The floors between the isolation layers were considered as standard floors, with the bottom layer being the isolation layer, followed by a standard floor, another isolation layer, another standard floor, and another isolation layer in sequence.

### Dynamic equilibrium equation

To establish the dynamic equilibrium equation for this type of structure, it is necessary to consider the mass, elastic characteristics, and forces acting on each component. Assuming that the entire structure can be viewed as an elastic single degree of freedom (SDOF) system, the following dynamic equilibrium equation can be used to describe this system: $ma + cv + kx = - F(t)$. Here, ${\text{m}}$ represents the mass of the system, *a* is the acceleration, *c* is the damping coefficient, *v* is the velocity, *k* is the stiffness coefficient, *x* is the displacement of the structure, and $F(t)$ is the force applied to the structure. For this seismic isolation device, since the entire structure is in the vertical direction, the force acting on it can be considered as perpendicular to the ground. Additionally, since there are isolation layers between each standard floor, each standard floor and isolation layer can be viewed as an individual SDOF system, and their coupling can be neglected. Therefore, a dynamic equilibrium equation can be established as follows:

$$\left\{ {\begin{array}{ll} {m_{1} a_{1} + c_{1} v_{1} + k_{1} x_{1} = - F_{1} (t)} \\ {m_{2} a_{2} + c_{2} v_{2} + k_{2} (x_{2} - x_{1} ) = - F_{2} (t)} \\ {m_{3} a_{3} + c_{3} v_{3} + k_{3} (x_{3} - x_{2} ) = - F_{3} (t)} \\ {m_{4} a_{4} + c_{4} v_{4} + k_{4} (x_{4} - x_{3} ) = - F_{4} (t)} \\ {m_{5} a_{5} + c_{5} v_{5} + k_{5} (x_{5} - x_{4} ) = - F_{5} (t)} \\ {m_{6} a_{6} + c_{6} v_{6} + k_{6} (x_{6} - x_{5} ) = - F_{6} (t)} \\ \end{array} } \right.$$

In this context, the variable $m_{i}$ represents the mass of each floor, $a_{i}$ represents the acceleration of each floor, $v_{i}$ represents the velocity of each floor, $x_{i}$ represents the displacement of each floor, and $F_{i} (t)$ represents the applied force on each floor. In the given statement, $k_{1}$, $k_{3}$, $k_{5}$ represent the stiffness coefficients of the structure, while $k_{2}$,$k_{4}$ and $k_{6}$ represent the stiffness coefficients of the isolation layer, and $x_{1}$, $x_{3} - x_{2}$, and $x_{5} - x_{4}$ represent the displacement differences of the isolation layer, indicating the relative displacement between the upper and lower layers. This paragraph describes that by solving the dynamic balance equations for each layer, the dynamic balance equation for the entire structure can be obtained. This equation provides the theoretical basis for the subsequent finite element analysis.

## Numerical model

### Engineering overview

**Figure 2:**
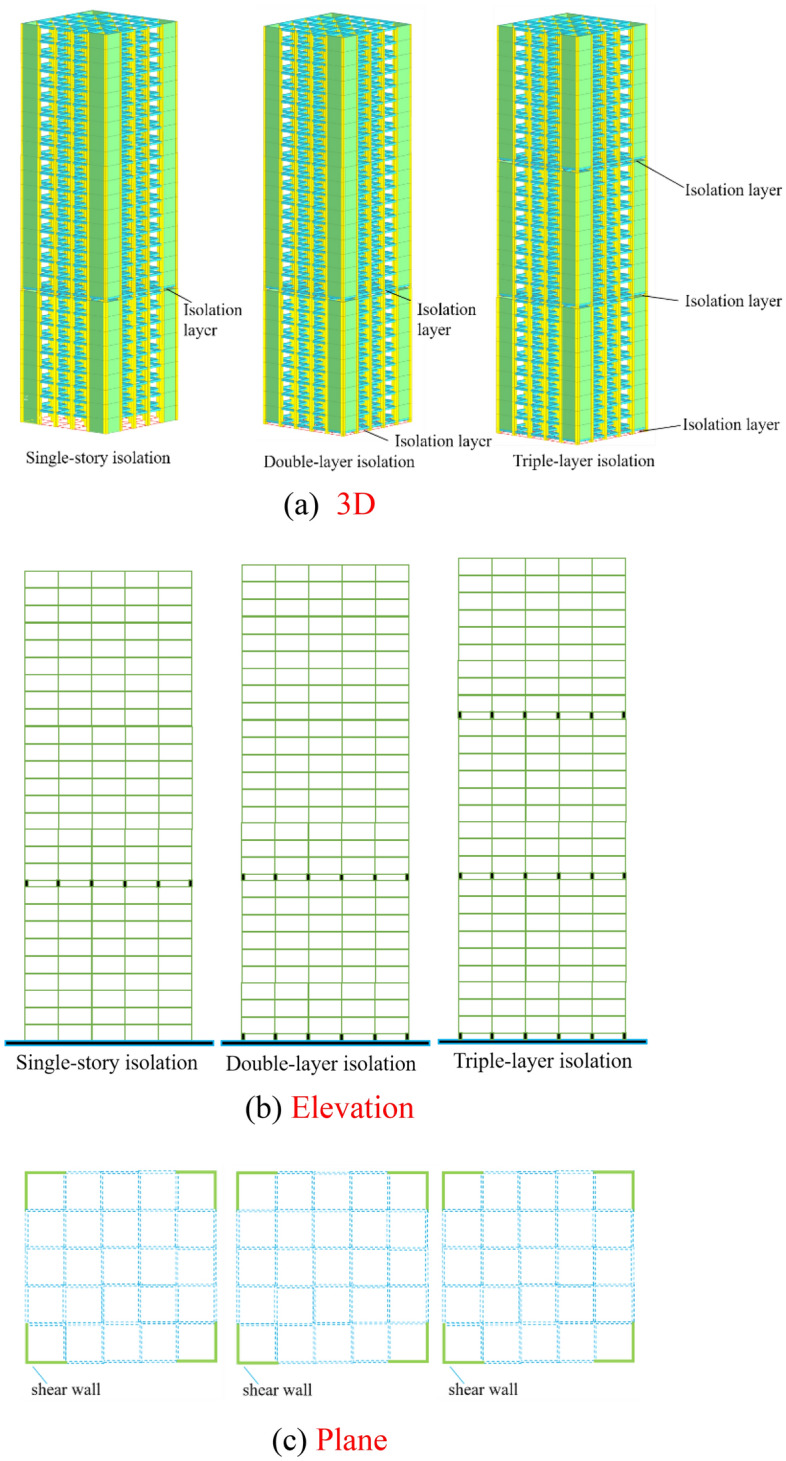
Schematic diagrams of single-story isolation, double-layer isolation, and triple-layer isolation structures. (**a**) 3D; (b) Elevation; (**c**) Plane.

According to China’s seismic design code^14^, this study concerns a 27-story reinforced concrete frame-shear wall structure with a seismic fortification intensity of 8 degrees on a class II site. The design earthquake group is the second group. The layout of the project can be seen in Fig. 2, with a square-shaped structure that has a total height of 108 m, a side length of 30 m, and a story height of 4.0 m. The concrete is of C40 grade, with a minimum design strength of 40 megapascals (MPa). Longitudinal reinforcing steel employs HRB400 grade, with a minimum yield strength of 400 MPa, while the tie reinforcement is chosen to be of HRB335 grade, with a minimum yield strength of 335 MPa. Other material properties are set to default values. The cross-sectional dimensions of the frame columns are 900 mm × 900 mm for floors 1–9, 700 mm × 700 mm for floors 10–18, and 600 mm × 600 mm for floors 19–27. The cross-sectional dimensions of the frame beams are 500 mm × 300 mm, the floor thickness is 100 mm, and the shear wall thickness is 400 mm. The structure is divided into three groups based on the number of layers of seismic isolation, including single-story isolation, double-layer isolation, and triple-layer isolation, as shown in Fig. 2.

### Establishment of models

**Figure 3:**
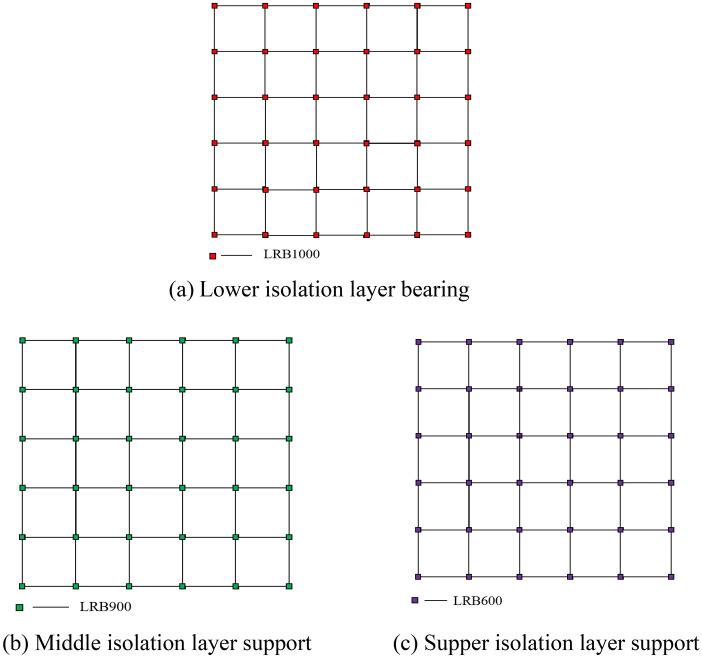
Layout of isolation bearings. (**a**) Lower isolation layer bearing; (**b**) Middle isolation layer support; (**c**) Supper isolation layer support.

The models of high-rise building structures with single-story isolation, double-layer isolation, and triple-layer isolation were established using Abaqus software. The triple-layer isolation device was implemented on the 1st, 9th, and 18th floors. In order to accommodate the diverse influences of ground motion on different building floors, various types of isolation devices exhibit distinct stiffness and damping characteristics. By employing a variety of isolation devices on different floors, it becomes possible to effectively tailor the dynamic response of the building structure. Isolation devices with lower stiffness contribute to reducing the natural frequency of the building, consequently mitigating structural vibrations under seismic excitations. Different floors may experience varying loads and stresses. Typically, at the base level, the entire weight of the building needs to be supported, necessitating the use of isolation devices with higher stiffness. However, at higher floors, loads are generally lower, allowing for the implementation of isolation devices with lower stiffness to reduce structural rigidity. Furthermore, based on the calculation of the load-bearing area using seismic isolation bearings, the models for lead-core rubber bearings have been determined as LRB1000, LRB900, and LRB600, while simultaneously ensuring their centroid aligns with the centroid of the column. Isolation bearings with diameters of 1000, 900, and 600 were used in the lower, middle, and upper isolation layers, respectively. The layout of isolation bearings can be observed in Fig. 3. The design parameters of isolation bearings are presented in Table 1, and two structural systems, namely double-layer isolation and single-story isolation, were established. The lower isolation layer of the double-layer isolation device was placed on the 1st floor, with LRB1000 isolation bearings used throughout. In the middle isolation layer, located on the 9th floor, all isolation bearings had a diameter of 900 and used LRB900. The isolation layer of the single-story isolation structure was also located on the 9th floor, and all isolation bearings used LRB900. According to the seismic design code^14^, the equivalent horizontal stiffness and equivalent damping ratio of the lead rubber bearings were determined assuming a shear deformation of 250% under rare earthquake conditions.

**Table 1:** Performance parameters of lead-rubber bearings.

Model	Effective diameter /mm	Rubber thickness /mm	Pre-yield stiffness /(kN/m)	100% horizontal shear deformation /(kN/m)	250% horizontal shear deformation /(kN/m)	Vertical stiffness /(kN/m)	Yield force /kN
LRB1000	1000	204	23,720	1820	–	12,504	203
LRB900	900	184	21,400	1650	–	11,144	141
LRB600	600	122	14,350	1100	–	6796	63

### Selection of earthquake waves

**Figure 4:**
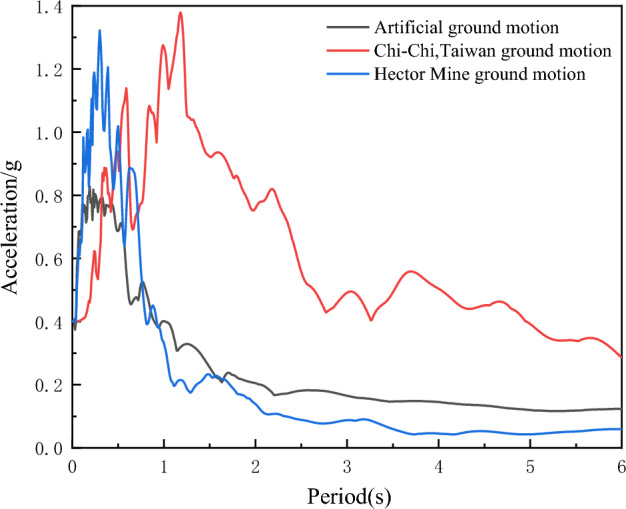
Acceleration response spectrum.

The seismic fortification intensity in the region is eight degrees. According to the requirements of the "Seismic Design Code", three seismic waves, including two natural waves and one artificial wave, were inputted into the three isolation structures. The earthquake waves need to be selected to have a main period that is close to the building site's predominant period. In addition, the earthquake waves must meet the requirements of the three elements of seismic activity: spectral characteristics, amplitude, and the duration of the earthquake acceleration time history. The acceleration response spectra of the three earthquake waves are shown in Fig. 4.

## Time history analysis

By employing the finite element software Abaqus, a high-rise structural shear wall system was constructed. Selected seismic data was input into the model and utilized as the excitation for a time history analysis. This typically involves coupling the seismic waves with the model and simulating the temporal evolution of seismic events.

Modal analysis was performed on three structural systems, including single-story isolation, double-layer isolation, and triple-layer isolation, as shown in Table 2 for the first six natural periods. The use of any of these isolation systems can extend the natural periods of the structure. Specifically, the triple-layer isolation system has a longer natural period than the double-layer isolation system, while the double-layer isolation system has a longer natural period than the single-story isolation system. This is because the triple-layer isolation system has an additional isolation layer above the double-layer isolation system, which increases the structural flexibility and further extends the natural period. Compared with the single-story isolation and double-layer isolation structures, the higher modes of vibration (4th, 5th, and 6th) of the double-layer isolation device are significantly increased, indicating that the natural vibration characteristics of the triple-layer isolation device are similar to those of the single-story isolation and double-layer isolation devices, all of which can effectively reduce the seismic response of the upper structure. This suggests that the higher modes of vibration of the triple-layer isolation device are significantly extended, thereby improving its damping performance and reducing the likelihood of damage to the upper structure under seismic actions.

**Table 2:** The first six natural periods (in seconds) for three structural systems under rare ground motion.

Order of vibration mode	Single-story isolation	Double-layer isolation	Triple-layer isolation
1	4.7044	5.5705	5.9853
2	4.7043	5.5705	5.9852
3	3.6062	4.4250	4.8422
4	1.2273	1.7296	2.0829
5	1.2273	1.7296	2.0828
6	0.9077	1.4305	1.8628

## The results of tensile and compressive stresses of isolation bearings

**Figure 5:**
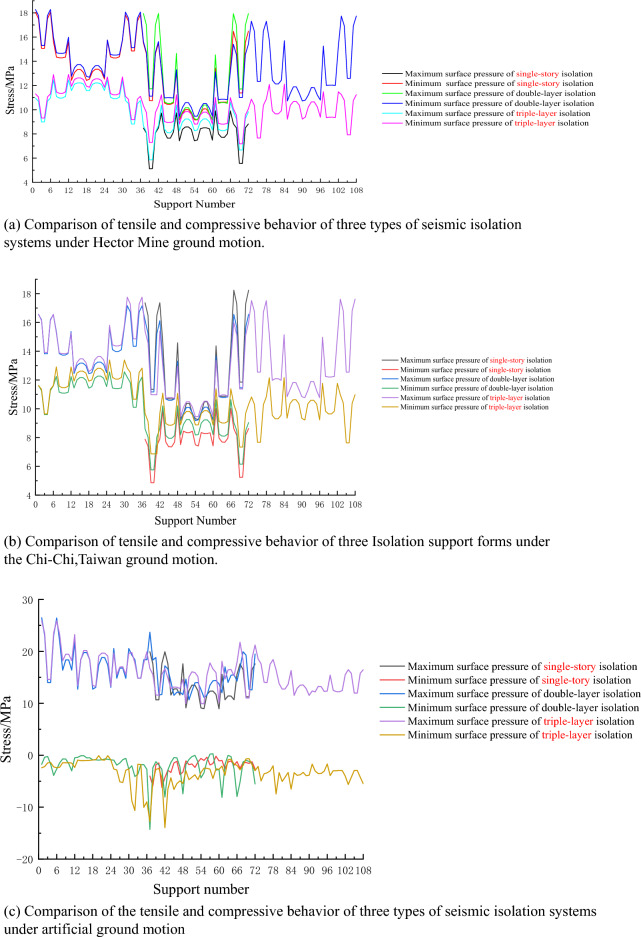
Comparison of tensile and compressive behavior of seismic isolation systems in supports. (**a**) Under Hector Mine ground motion; (**b**) under Chi-Chi, Taiwan ground motion; (**c**) under artificial ground motion.

According to the "Seismic Design Code for Buildings", the tensile stress of rubber isolation bearings should not exceed 1 MPa, and the compressive stress should not exceed 30 MPa during rare earthquakes. Under the effect of three seismic waves, the maximum compressive stress of the isolation bearings in the structure did not exceed 30 MPa, meeting the requirements of the code. The stress results of the isolation bearings are shown in Table 3. The isolation bearings with Numbers 37–72 are for the single-story isolation device, the bearings with Numbers 1–72 are for the double-layer isolation device, and the bearings with Numbers 1–108 are for the triple-layer isolation device, as shown in Fig. 5. Use Origin (2019) software to analyze and process experimental data. By comparing the minimum surface pressure of the three types of isolation devices with the bearing Numbers 1–36 and 37–72, it is found that the differences are not significant. The maximum surface pressure of the single-story isolation device is the largest, while that of the triple-layer isolation device is the smallest. The maximum and minimum surface pressures of the isolation bearings with Numbers 71–108 in the triple-layer isolation device are much smaller than the code requirement. Among the three types of isolation devices, tensile stress occurred under artificial waves. By comparing and analyzing the results, it is found that single-story and double-layer isolation devices have larger tensile stress, while the absolute value of tensile stress of the triple-layer isolation device is much smaller than the other two types.

**Table 3:** Stress in seismic isolation supports.

Calculated values	Single-story isolation	Double-layer isolation	Triple-layer isolation
Maximum surface pressure (MPa)	19.93	26.48	25.90
Minimum surface pressure (MPa)	−6.19	−14.28	−13.95
Average minimum surface pressure (MPa)	4.6	5.95	5.79
Average maximum surface pressure (MPa)	12.74	14.24	14.17
Variance of minimum surface pressure (MPa)	1.61	3.99	3.42
Variance of maximum surface pressure (MPa)	9.76	8.73	7.81

According to Table 3, it can be observed that all three types of seismic isolation structures experience tensile stresses in their supports during rare seismic events. It has been confirmed that the seismic isolation supports experiencing tensile stresses are predominantly structural angle supports, and they represent a relatively small proportion. Among them, only one support in the triple-story isolation device exceeds the specified tensile stress limit of 1 MPa, while the single-layer seismic isolation and double-layer seismic isolation devices exceed the limit more frequently. The average minimum face pressure of the three devices is similar, but the variance of the minimum face pressure in the triple-layer seismic isolation structure is slightly higher than that in the interlayer seismic isolation structure and lower than that in the double-layer seismic isolation device. This indicates that the minimum face pressure dispersion of the seismic isolation supports in the triple-layer device is relatively small, and the coordination of the device with regard to tensile stresses in the supports is more appropriate. The minimum face pressure of the supports can be more easily controlled, and tensile stress is less likely to occur. The maximum face pressure of all three seismic isolation devices is less than 27 MPa, and the variance of the maximum face pressure in the triple-story isolation device is slightly lower than that in the other two seismic isolation devices, indicating a more uniform distribution of maximum face pressure in the seismic isolation supports of the triple-layer device.

## Analysis of seismic response of triple-layer isolation, single-story isolation, and double-layer isolation devices

### Comparative analysis of the effectiveness of different seismic isolation structures under rare seismic events

According to the "Code for Seismic Design"^14^, two actual earthquake records (Hector Mine wave, with a peak acceleration of 400 gal, and Chi-Chi, Taiwan wave, with a peak acceleration of 400 gal) and one artificial earthquake wave (ArtWave-RH3TG045, with a peak acceleration of 400 gal) were selected for seismic response analysis. The peak accelerations of the three earthquake waves were adjusted to 400 gal, which is equivalent to the peak acceleration corresponding to seismic intensity of 8 degrees under rare seismic events. The seismic response of the three structural systems under rare seismic events is shown in Table 4.

**Table 4:** Maximum seismic response of three structural systems under rare ground motion.

Type of earthquake motion		Maximum displacement of the lower isolation layer (cm)	Maximum displacement of the middle isolation layer (cm)	Maximum displacement of the upper isolation layer (cm)	Peak acceleration at the top level (gal)
	Single-story isolation	–	3.1	–	10.84
Chi-Chi, Taiwan	Double-layer isolation	6.1	2.0	–	7.07
	Triple-layer isolation	8.5	2.5	4.3	7.22
	Single-story isolation	–	5.5	–	7.39
Hector Mine	Double-layer isolation	4.6	5.6	–	7.08
	Triple-layer isolation	9.3	5.4	5.6	7.23
	Single-story isolation	–	2.6	–	205.73
ArtWave	Double-layer isolation	7.9	1.7	–	180.96
	Triple-layer isolation	16.5	4.2	4.8	297.82

Based on Table 4, it can be observed that under the effects of the Chi-Chi, Taiwan and Hector Mine ground motion, the peak accelerations of the triple-layer isolation device were 66.6% and 97.8% of the single-story isolated device, respectively. Under the artificial ground motion, the top-floor acceleration of the three-layer isolation device was slightly higher. During rare earthquakes, the average peak acceleration of the top floor of the triple-layer isolation device was smaller than that of the single-story and double-layer isolation devices, which effectively mitigates the transmission of seismic energy to the upper parts of the structure. Under the influence of the Chi-Chi, Taiwan and Hector Mine ground motion, the maximum displacement of the middle isolation layer of the triple-layer isolation device was 80.6% and 98.2% of the single-story isolation device, respectively. Under artificial ground motion, the maximum displacement of the middle isolation layer of the triple-layer isolation device was slightly higher than that of the middle isolation layer of the double-layer isolation device. Under these three ground motions, the maximum displacement of the upper isolation layer of the triple-layer isolation device was much smaller than that of the lower isolation layer. During rare ground motion, the average maximum displacement of the isolation layer of the triple-layer isolation device was smaller than that of the single-story and double-layer isolation devices.

### Inter-story displacement and inter-story shear force

According to seismic regulations, the allowable displacement values for the LRB1000 isolation bearing are 550 mm, LRB900 is 495 mm, and LRB600 is 330 mm. From Table 4, it can be seen that the maximum displacement values of the lower, middle, and upper isolation layers do not exceed the specified limits.

**Figure 6:**
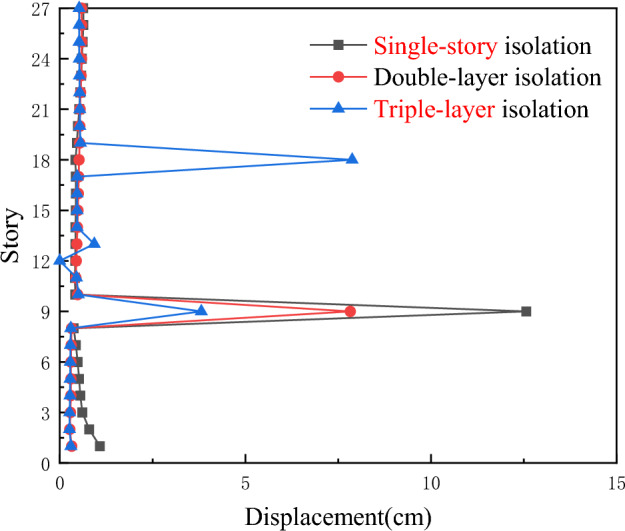
Comparison of inter-story displacements for three types of seismic isolation under Hector Mine ground motion.

As shown in Fig. 6, under the action of the Hector Mine earthquake wave, the inter-story drift of the triple-layer isolation system is smaller than that of the single-story isolation and the double-layer isolation layer. With the increase in the position of the middle isolation layer, the displacement of the middle isolation layer decreases, while the displacement of the inter-story isolation layer increases. The triple-layer isolation device can further reduce the inter-story drift. The displacements of the lower isolation layer and the middle isolation layer under this isolation system are smaller than those under the inter-story isolation and double-layer isolation systems.

**Figure 7:**
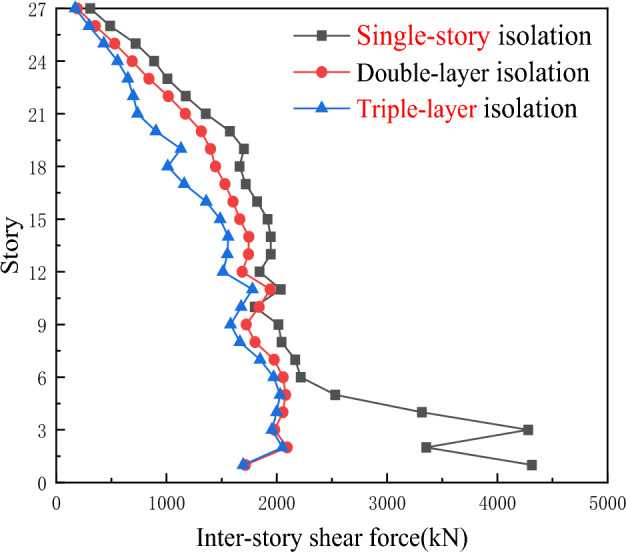
Comparison of inter-story shear forces for three types of seismic isolation under Hector Mine ground motion.

As shown in Fig. 7, compared with the single-story isolation device, the floor shear force of the double-layer isolation structure is significantly reduced, while the floor shear force of the triple-layer isolation device is better than that of the double-layer isolation device. This not only indicates that the setting of the isolation layer reduces the section force of the structural floor shear components to some extent and improves the seismic performance of the structure, but also shows that the shear resistance of the floors in the triple-layer isolation device continuously increases with the increase of the number of layers.

**Figure 8:**
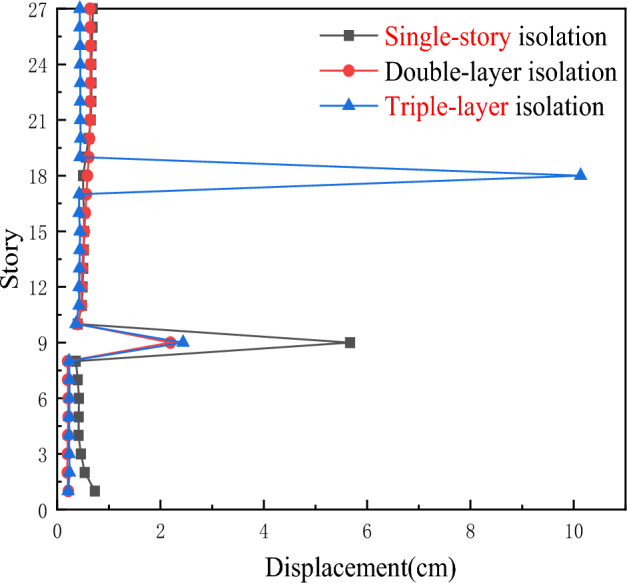
Comparison of inter-story displacements for three types of seismic isolation under Chi-Chi, Taiwan ground motion.

According to Fig. 8, under the action of Chi-Chi, Taiwan earthquake waves, the maximum inter-story displacement of the three-layer isolation system is reduced to varying degrees compared with the single-story isolation and double-layer isolation devices, and all isolation layers undergo sudden changes. The inter-story displacement of the other layers is also significantly reduced. Therefore, it can be concluded that the triple-layer isolation device has a better seismic isolation effect.

**Figure 9:**
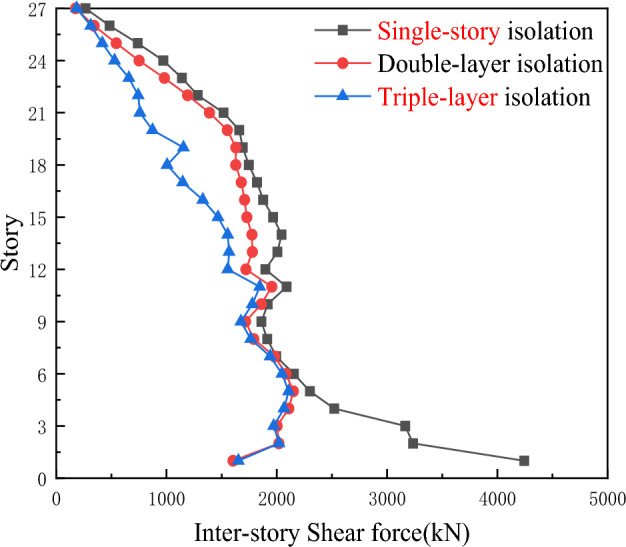
Comparison of inter-story shear forces for three types of seismic isolation under Chi-Chi, Taiwan ground motion.

As shown in Fig. 9, under the action of Chi-Chi, Taiwan earthquake waves, the inter-story shear forces of the single-story isolation and double-layer isolation devices are much greater than those of the triple-layer isolation device. Therefore, the triple-layer isolation device can exhibit a better seismic isolation effect, with the double-layer isolation being the second best in terms of effectiveness.

**Figure 10:**
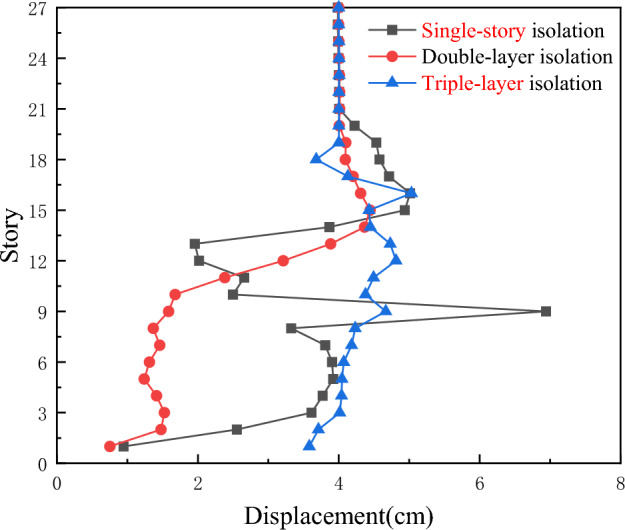
Comparison of inter-story displacements for three types of seismic isolation under artificial ground motion.

As shown in Fig. 10, although the inter-story displacement of the three isolation layers is not significantly different under the action of artificial waves, as the building height increases, the inter-story displacement of the triple-layer isolation device becomes relatively smaller compared to the other two types of isolation structures.

**Figure 11:**
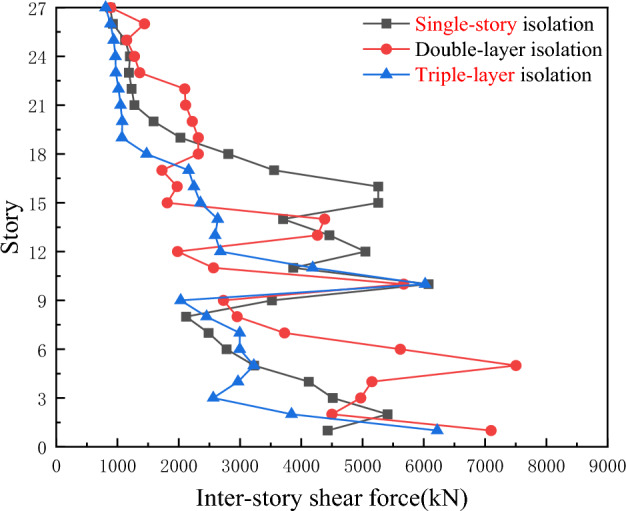
Comparison of inter-story shear forces for three types of seismic isolation under artificial ground motion.

As shown in Fig. 11, under the action of artificial waves, especially rare earthquake events, the shear resistance of the triple-layer isolation structure is stronger than that of the single-story isolation and double-layer isolation devices below the 6th floor and above the 18th floor, particularly in the upper part of the overall structure. Therefore, the triple-layer isolation device has a better isolation effect and is particularly suitable for ultra-high-rise buildings.

### Floor response acceleration

**Figure 12:**
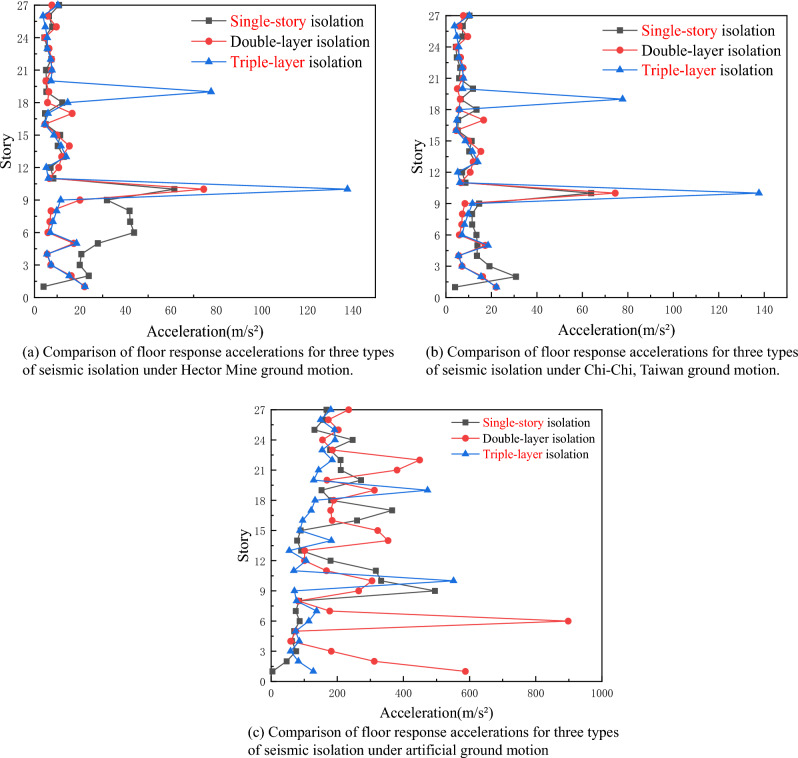
Floor response acceleration. (**a**) Under Hector Mine ground motion; (**b**) under Chi-Chi, Taiwan ground motion; (**c**) under artificial ground motion.

According to Fig. 12, it is observed that the acceleration response of the seismic isolation device is significantly reduced after inputting three seismic waves. The dramatic increase in acceleration for a triple-layer seismic isolation structure mainly occurs within the isolation layer, which indicates that as the number of isolation layers increases, the seismic energy transmitted to the building can be continuously dissipated. The acceleration of the floors in the triple-layer isolation device is more stable compared to the single-story isolation and double-layer isolation devices, and the acceleration difference among the floors in the triple-story seismic isolation system is small.

Under the influence of the Hector Mine and Chi-Chi, Taiwan waves, the floor response acceleration of the three types of isolation forms mainly concentrates near the isolation layer. Compared with the other two isolation structures, the floor response acceleration of the triple-layer isolation structure is not significantly different in the upper part of the entire floor, but in the middle and lower parts, the floor response acceleration of the triple-story isolation device is smaller. Therefore, it can be considered that the triple-layer isolation device can more significantly reduce the floor response acceleration in the middle and lower parts of the entire building floor.

Under artificial wave excitation, the floor response acceleration of the three types of isolation devices mainly concentrates near the isolation layer. However, overall, the floor response acceleration of the triple-layer isolation device is always the smallest.

## Overturning moment

**Figure 13:**
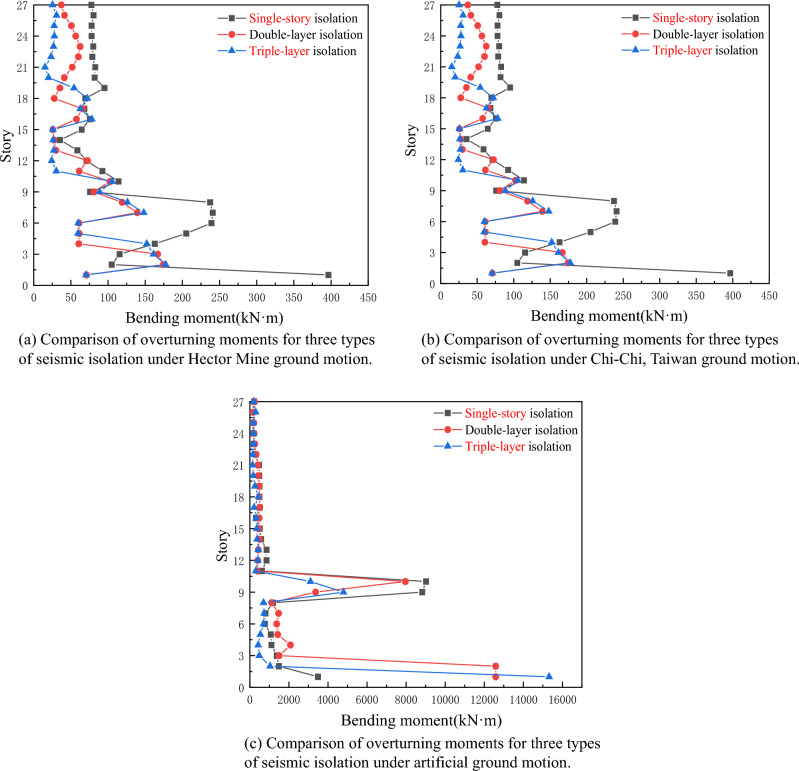
Overturning moment. (**a**) under Hector Mine ground motion; (**b**) under Chi-Chi, Taiwan Seismic ground motion; (**c**) under artificial ground motion.

Based on Fig. 13, after inputting three seismic waves and comparing the internal forces of the three seismic isolation devices, it is evident that the overturning moment of the triple-layer seismic isolation device is significantly reduced. Compared to single-story isolation and double-layer isolation devices, the triple-layer isolation structure is more effective in reducing internal forces in ultra-high-rise buildings, and its advantage becomes more prominent with an increasing number of floors, while also reducing the structural deformation.

Under the action of Hector Mine and Chi-Chi, Taiwan waves, the maximum overturning moment of the three seismic isolation devices is concentrated in the middle and lower parts of the building structure, especially in the location of the seismic isolation layer. Therefore, the arrangement of the seismic isolation layer and the selection of the seismic isolation support should be given special attention. As mentioned above, the overturning moment in the lower part of the triple-layer isolation device is smaller compared to the other two types of isolation devices, demonstrating its significant advantages.

Under artificial waves, the difference in overturning moment among the three seismic isolation devices is mainly reflected in the location of the seismic isolation layer. The maximum overturning moment of the triple-story seismic isolation device is near the lower seismic isolation layer, followed by the double-layer isolation device, and the single-story isolation device has the smallest overturning moment. However, as the number of seismic isolation layers increases, the overturning moment of the triple-layer isolation structure continues to decrease.

Based on our comprehensive analysis results and the seismic isolation bearing characteristics listed in the Table 1, it is evident that the triple-layer isolation device exhibits outstanding performance under rare seismic events. Firstly, the triple-layer isolation device demonstrates a significant advantage in controlling inter-story displacements, contributing to the overall stability of the building. Furthermore, through comparative analysis, we have observed that the triple-layer isolation device also performs better in reducing inter-story shear forces and floor accelerations. Finally, the results of overturning moment analysis further endorse the superiority of the triple-layer isolation device, indicating its higher stability and reliability in resisting lateral loads during rare seismic events. Consequently, taking into account various performance aspects under rare seismic conditions, we firmly believe that the triple-layer isolation device is the optimal choice, providing the best seismic resistance and structural safety for the building.

## Conclusions

Modal analysis of the structural system shows that the triple-layer seismic isolation device can further prolong the natural vibration period of the structure compared to the interlayer and double-layer seismic isolation devices.

The minimum face pressure average for the triple-layer isolated structure is 5.79 MPa, for the double-layer isolation is 5.95 MPa, and for the single-story isolation is 4.60 MPa. The minimum face pressure dispersion of the seismic isolation bearings in the triple-layer seismic isolation device is relatively small, and the coordination of the device with respect to the tensile stress of the bearings is moderate. The minimum face pressure of the bearings is relatively easy to control, and tensile stress is not easily generated. The maximum face pressure variance of the triple-layer isolated structure is less than 11% compared to the double-story isolation, and the maximum face pressure variance of the triple-story isolation device is less than 20% compared to the single-story isolation. The variance of the maximum face pressure of the triple-layer seismic isolation device is slightly smaller than that of the other two types of seismic isolation devices, indicating that the maximum face pressure distribution of the bearings in the triple-layer seismic isolation device is more uniform. Therefore, in comparison to the inter-story isolation structure and the double-layer isolation structure, the three-layer isolation device exhibit superior performance in terms of isolator compressive and tensile stresses.

In comparison to the single-story isolation device and the double-layer isolation device, the triple-layer isolation system can significantly reduce inter-story displacement, inter-story shear force, and overturning moment, particularly in the upper-middle part of the super high-rise building under rare seismic events.

Under rare seismic events, the triple-layer seismic isolation structure can effectively reduce the acceleration of the floors in the super high-rise building, and it is more stable compared to the other two types of seismic isolation devices.

## Data Availability

The datasets used and/or analysed during the current study available from the corresponding author on reasonable request.

## References

[1] Ou JP, Wu PS, Guan XCH (2021). Resilient structures with large displacement friction pendulum system and multi-layer isolation system. J. Disast. Prev. Mitig. Eng..

[2] Wang XQ (2007). Study on a new type of SMA-rubber bearing and its segmented isolation application in high-rise buildings (Doctoral dissertation). Liaoning Tech. Univ. China.

[3] Gao JP, Luo D, Pan YY (2011). Optimization of passive control parameters of segmented isolation system for high-rise buildings with transition layer. J. East China Jiaotong Univ..

[4] Wu QY, He WCH, Zhu HP (2020). Effect of mixed passive control systems under different frequency domain seismic excitations on reduction and isolation. J. Lanzhou Univ. Technol..

[5] Ryan KL, Earl CL (2010). Analysis and design of inter-story isolation systems with nonlinear devices. J. Earthq. Eng..

[6] Ziyaefar M, Noguchi H (1998). Partial mass isolation in tall buildings. Earthq. Eng. Struct. D.

[7] Tso-Chien P, Shih-Fu L, Wei C (1995). seismic response of segmental building. Earthq. Eng. Struct. D.

[8] Charmpis DC, Komodromos P, Phocas MC (2012). Optimized earthquake response of multi-storey buildings with seismic isolation at various elevations. Earthq Eng Struct D..

[9] Skandalos, K. & Tesfamariam, S. *Multi-story Isolated Buildings: A Multi-objective Study*. (2022)

[10] Du H, Wang Y, Han M, Ibarra LF (2021). Experimental seismic performance of a base-isolated building with displacement limiters. Eng. Struct..

[11] Han M, Wang Y, Du H, Chu X, Cui M, Meng L (2021). Base-isolated steel structure with spring limiters under near-fault earthquakes: Experiment. Earthq. Struct..

[12] Deringöl AH, Güneyisi EM (2021). Influence of nonlinear fluid viscous dampers in controlling the seismic response of the base-isolated buildings. Structures.

[13] ABAQUS. *Version 16.4*. (Dassault Aircraft Company, 2016).

[14] Ministry of Housing and Urban-Rural Development of the People’s Republic of China (2016). GB50011–2010 Code for seismic design of buildings.

